# AXL kinase-mediated astrocytic phagocytosis modulates outcomes of traumatic brain injury

**DOI:** 10.1186/s12974-021-02201-3

**Published:** 2021-07-07

**Authors:** Hang Zhou, Libin Hu, Jianru Li, Wu Ruan, Yang Cao, Jianfeng Zhuang, Hangzhe Xu, Yucong Peng, Zhongyuan Zhang, Chaoran Xu, Qian Yu, Yin Li, Zhangqi Dou, Junwen Hu, Xinyan Wu, Xiaobo Yu, Chi Gu, Shenglong Cao, Feng Yan, Gao Chen

**Affiliations:** 1 Department of Neurosurgery, Second Affiliated Hospital, School of Medicine, Zhejiang University, Jiefang Road88th, Hangzhou, 310016 China; 2 Department of Burn and Plastic Surgery, Children’s Hospital, Zhejiang University School of Medicine, No. 3333 Binsheng Road, Zhejiang, 310052 Hangzhou China; 3 Department of Neurosurgery, Children’s Hospital, Zhejiang University School of Medicine, No. 3333 Binsheng Road, Zhejiang, 310052 Hangzhou China

**Keywords:** AXL kinase, Reactive astrocytes, Phagocytosis, Traumatic brain injury

## Abstract

**Background:**

Complex changes in the brain microenvironment following traumatic brain injury (TBI) can cause neurological impairments for which there are few efficacious therapeutic interventions. The reactivity of astrocytes is one of the keys to microenvironmental changes, such as neuroinflammation, but its role and the molecular mechanisms that underpin it remain unclear.

**Methods:**

Male C57BL/6J mice were subjected to the controlled cortical impact (CCI) to develop a TBI model. The specific ligand of AXL receptor tyrosine kinase (AXL), recombinant mouse growth arrest-specific 6 (rmGas6) was intracerebroventricularly administered, and selective AXL antagonist R428 was intraperitoneally applied at 30 min post-modeling separately. Post-TBI assessments included neurobehavioral assessments, transmission electron microscopy, immunohistochemistry, and western blotting. Real-time polymerase chain reaction (RT-PCR), siRNA transfection, and flow cytometry were performed for mechanism assessments in primary cultured astrocytes.

**Results:**

AXL is upregulated mainly in astrocytes after TBI and promotes astrocytes switching to a phenotype that exhibits the capability of ingesting degenerated neurons or debris. As a result, this astrocytic transformation promotes the limitation of neuroinflammation and recovery of neurological dysfunction. Pharmacological inhibition of AXL in astrocytes significantly decreased astrocytic phagocytosis both in vivo and in primary astrocyte cultures, in contrast to the effect of treatment with the rmGas6. AXL activates the signal transducer and activator of the transcription 1 (STAT1) pathway thereby further upregulating ATP-binding cassette transporter 1 (ABCA1). Moreover, the supernatant from GAS6-depleted BV2 cells induced limited enhancement of astrocytic phagocytosis in vitro.

**Conclusion:**

Our work establishes the role of AXL in the transformation of astrocytes to a phagocytic phenotype via the AXL/STAT1/ABCA1 pathway which contributes to the separation of healthy brain tissue from injury-induced cell debris, further ameliorating neuroinflammation and neurological impairments after TBI. Collectively, our findings provide a potential therapeutic target for TBI.

**Supplementary Information:**

The online version contains supplementary material available at 10.1186/s12974-021-02201-3.

## Background

Traumatic brain injury (TBI) is a worldwide problem for which there are few efficacious therapeutic interventions [1]. The initiation of mechanical damage and subsequent injurious biochemical cascades induce cerebral microenvironment changes that underlie many neurological and psychiatric function disorders observed in TBI patients. Under brain-injured conditions, microglia and astrocytes become reactive, which were well recognized as major regulators in immune modulation and microenvironmental homeostasis [2].

Microglia are resident immune cells involved in brain homeostasis surveillance [3] that have a sensitive response and rapid activation within 1 hour after TBI. This is followed by reactive astrocytes, which are also resident brain cells that can dramatically transform their phenotype as a result of brain environment changes [4, 5]. Following the activation of microglia and astrocytes, an extensive and lasting immune response is sustained through a complex cascade of events, such as excitotoxicity, free radical generation, and neuroinflammation [2]. However, despite the recognition of the diverse actions of microglia in response to TBI, relatively few mechanisms exist on altering the capacity of astrocytes to mount an appropriate response to TBI. Recent evidence has shown that unhealthy astrocytes may release toxic factors that damage the surrounding tissues [6]; in contrast, reactive astrocytes form a characteristic phenotype that protects normal tissue by preventing and limiting the infiltration of inflammatory cells or molecules after injury [7, 8]. Thus, the potential mechanisms of astrocytic reactivation and induction of neuroprotective effects are not fully understood. Regarding glial activation under pathological conditions, it remains unclear how these two types of glial cells interact.

After brain injury, widespread neuronal cell death causes the collection of debris and unique initial environmental changes within the damaged area. Rapid, consistent engulfing, and clearance of dead cells or debris is critical for the prevention of secondary injury and helps to remodel the brain microenvironment, otherwise elevating the risk of neurological disorders [9, 10]. The main professional phagocytes, microglia, have been well characterized [10, 11]. However, growing evidence indicates that non-professional phagocytes also contribute to this process [12–14]. Astrocytes have been shown to express genes enriched in engulfment pathways such as TAM receptors, draper/Megf10, and ABCA1 in gene profiling studies [14, 15]. Additionally, several previous studies have reported the presence of apoptotic neurons and debris in astrocytes after brain injury [16, 17]. Although accumulating evidence suggests the phagocytic role of astrocytes, astrocytic phagocytosis still receives limited attention and the mechanisms are unknown, especially in acute damage such as TBI.

The tyrosine kinases AXL is one of the receptors activated by the binding of PtdSer-expressing apoptotic cells under its adapter protein GAS6 [18, 19]. In a preclinical model of intracerebral hemorrhage, AXL has been shown to enhance macrophage phagocytosis and hematoma clearance [20]. Upon activation, AXL not only facilitates the action of phagocytic ability, but also triggers anti-inflammatory responses via Toll-like receptor (TLR) signaling associated with STAT1 and STAT3 [21, 22]. AXL has been suggested as a potential therapeutic target in several diseases such as Alzheimer's disease and malignant tumors [23]. However, whether AXL kinase is associated with astrocytic phagocytosis and how it affects the pathological process of TBI remains to be determined.

Therefore, this study aims to indicate that reactive astrocytes transformed into a phagocytic phenotype. This was mediated by AXL, in which the natural ligand GAS6 was microglia-derived after TBI. The phagocytic astrocytes acted to limit interaction between injured cells or debris and healthy tissues, and attenuate neurological outcomes. This finding provides a novel therapeutic target for TBI and its complications.

## Materials and methods

### Animals

Male C57BL/6 mice (8-week-old, 23–25 g) were obtained from SLAC Laboratory Animal Company Limited (Shanghai, China) and housed individually in home cages with a 12-h light/dark cycle with food and water ad libitum. The animal protocol was approved by the Institutional Ethics Committee of the Second Affiliated Hospital, Zhejiang University School of Medicine. All procedures followed the ARRIVE and RIGOR guidelines.

### Controlled cortical impact

Animals were anesthetized using pentobarbital sodium (40 mg/kg, intraperitoneally). The details to produce the controlled cortical impact (CCI) injury have been described previously [24, 25]. Briefly, the mouse was mounted on a stereotaxic frame using ear bars and an incisor bar. The craniotomy was located approximately midway between the bregma and lambda on the left side, with the medial edge of the craniotomy 1 mm lateral to the midline. The skull disk was then removed without disturbing the dura. The CCI was performed perpendicular to the brain surface using a PinPoint™ Precision Cortical Impactor (Cary, NC, USA). The parameters were set as 2.5-mm-diameter impact tip, 3.0-m/s impact velocity, 100-ms impact duration time, and 1-mm displacement of the brain. The core body temperature was maintained at 36.0–36.5 °C using a rectal thermometer coupled to a heating pad. After the injury, the bone flap was immediately replaced, sealed, and the scalp was sutured closed. Sham-operated animals received identical surgical procedures without CCI. All animals were allowed to completely recover from the anesthesia exposure in a heated chamber before returning to their home cages.

### Behavioral assessments

After the intervention, mice were subjected to behavioral tests, including the modified neurological severity score (mNSS) (Supplemental table 1) [26, 27], tail suspension test (TST) [28, 29], and motor coordination (rotarod) [28], as previously described.

Motor function was assessed using the rotarod. Acclimation (4 days before injury) and testing (1–2 days before injury; 1, 3, and 7 days post injury) were performed 2–3 h prior to the dark phase of the photoperiod. Testing comprised 3 trials in which mice were placed on the rod at a speed of 10 rpm with 0.2-rpm/s acceleration. Trials 1 and 2 were performed back-to-back to prevent associations between falling and returning to the home cage, and mice were given a 10 min rest between trials 2 and 3. The average time spent on the rod before falling for the best two trials is reported. Baseline motor coordination was established as time spent on the rotarod 1 d before the injury.

Depressive-like behavior was assessed using the tail suspension test (TST). Briefly, mice were suspended by their tails. Immobility was recorded for 10 min. Results are expressed as time spent immobile.

### Drug administration

The specific AXL antagonist R428 (MedChemExpress, Monmouth Junction, NJ, USA) was injected intraperitoneally at 125 mg/kg to mice 30 min after CCI. Recombinant mouse Gas6 protein (rmGas6; 0.4 mg/kg, R&D Systems, Minneapolis, MN, USA) was dissolved in saline and delivered into the ipsilateral ventricle (1.0 mm lateral of the bregma, 0.45 mm lateral to the midline, and 2.5 mm deep) 30 min after CCI, with a total volume of 4 μL at a rate of 0.5 μL/min [23, 30].

### Fluoro-Jade C staining

After immunofluorescence staining, the Fluoro-Jade C (FJ) staining kit (Biosensis, Thebarton, South Australia USA) was used to identify degenerating neurons and debris [24]. Sections were dried at 50–60 °C for 15 min. Slides were immersed in 1% sodium hydroxide in 80% ethanol for 5 min, followed by rinsing for 2 min in 70% ethanol, then 2 min in distilled water. Slides were incubated in 0.06% potassium permanganate solution for 5 min with gentle shaking. Afterward, the sections were incubated in 0.0002% FJ staining buffer for 10 min in the dark. Slides were rinsed three times with distilled water for 1 min. The water was then drained and the slides were dried, cleared in xylene for 1 min, and coverslipped with DPX (Sigma, St. Louis, MO, USA). Images were taken under a fluorescence microscope (Lecia, DM550, Germany), and degenerating neurons were counted in six fields (200×) per section.

### Transmission electron microscopy

Mice were sacrificed and perfused with 0.01 M cold PBS and 4% paraformaldehyde (PFA). Tissues were processed as previously described [30]. Briefly, approximately 1 mm^3^ blocks of cortex were immersed in 2.5% glutaraldehyde at 4 °C overnight, followed by post-fixation in 1% osmium tetroxide for 1 h. Then, the samples were stained with 2% uranyl acetate and dehydrated in graded ethanol. After embedding in 100% resin overnight, the samples were cut into 100 nm sections and stained with 2% uranyl acetate and lead citrate. TEM images were captured with a Philips Tecnai 10 TEM in Zhejiang University and analyzed using Image J.

### Cell culture

Dissociated cerebral cortical cells from postnatal 0–1 mice were plated in T-75 flasks at a density of one mouse per flask (Corning Costar, NY, USA) precoated with poly-d-lysine (50 μg/mL; Sigma). Cultures were purified by shaking for 14–16 h on an orbital shaker at 250 rpm at 37 °C after 8–10 days, and astrocyte cultures were confirmed to contain very little microglia by flow cytometry (Supplemental Fig 2). Astrocytes were re-plated in 6-well plates with DMEM contained 10% FBS, 100 U/mL penicillin, and 100 μg/mL streptomycin at a density of 25,000 cells cm^−2^ [31].

For primary neurons, E18 mouse cerebral cortices were dissociated and plated in 6-well plates coated with PDL at a density of 50,000 cells cm^−2^. The culture medium Neurobasal (Gibco, Marylan, USA) supplemented with 2% B-27 (v/v) (Gibco), 2 mM glutamine (Gibco), 100 U/mL penicillin, and 100 mg/mL streptomycin was replaced every 3–4 days [31].

The BV2 microglial cell line was incubated overnight in DMEM high glucose supplemented with 10% FBS, 100 U/mL penicillin, and 100 μg/mL streptomycin. The cells were cultured in a serum-free medium under stimulation.

### In vitro TBI model and drug administration

The in vitro model of TBI was conducted by manual scratch according to previous studies [32, 33]. rmGas6 was dissolved in saline to 400 ng/mL, the STAT1 specific inhibitor fludarabine (1 μM, MCE, USA) treated for 1 h prior to rmGas6 treatment. A total of 1 μM R428 (diluted in 0.1% DMSO) or DMSO control was added to the cells for 1 h following media changes. Lipopolysaccharide (LPS, 100 ng/ml, Sigma)-stimulated BV2 supernatant (12 h) was collected for co-culture with primary neurons for 24 h as described previously [34].

### Transient cell transfection with siRNAs

BV2 cells or primary cultured astrocytes were transiently transfected with siRNA targeting GAS6 (100 nM), siRNA targeting AXL (100 nM), or control siRNA (Genomeditech, Shanghai, China) using 7.5 μL of Lipofectamine™ 3000 (Invitrogen, California, USA) according to the manufacturer’s protocol. The cells were incubated in Opti-MEM for 48 h, then changed to astrocyte culture and continually incubated for 24 h, and the GAS6 knockdown BV2 supernatant fluid was collected for the next step. The sequences used for GAS6 knockdown were sense 5-CGGAGUAUUUCUAUCCACGAU-3 and antisense 5-AUCGUGGAUAGAAAUACUCCG-3; the sequences used for AXL knockdown was sense 5-GGAGACCCGUUAUGGAGAATT-3 and antisense 5-UUCUCCAUAACGGGUCUCCTT-3.

### In vitro phagocytosis assay

The prepared injured neurons or debris were tagged with PKH26 red fluorescent dye (λex 551nm/λem 567nm, Sigma) in accordance with the manufacturer’s instructions, followed by incubation with primary astrocytes. After co-culture, the cells were washed three times with cold PBS, incubated with 0.25% EDTA-trypsin, suspended in DMEM with 10% serum, and washed twice with PBS. The cells were analyzed using FACS (BD Bioscience, New Jersey, USA). Free neurons or debris were excluded using forward and side-scattered plots (FSC and SSC, respectively) based on their physical properties. In the PE channel, the phagocytic astrocytes were distinguished with PKH26+ signal by comparing with negative control. For each sample, 10,000 events were collected and analyzed using FlowJo. Phagocytosis ability was identified as the percentage of fluorescent-positive cells multiplied by the geometric mean of fluorescence intensity [16].

### Immunofluorescence

Brains were post-fixed in 4% PFA overnight and dehydrated in 30% sucrose for 3 days after perfusion as described before [35]. Consecutive coronal frozen sections of 8-μm thickness were cut at 24-μm intervals and placed onto slides. The brain tissues were blocked and permeabilized with 10% BSA and 0.3% Triton X-100 for 1 h. Then, the slides were incubated at 4 °C overnight with primary antibody. After being washed with 0.01M PBS three times for10min each, the sections were incubated with secondary antibodies for 1 h at 37 °C in the dark. The sections were visualized by a fluorescence microscope (Olympus, Tokyo, Japan) and the images were analyzed using ImageJ software.

### Quantitative real-time PCR

RNA from cortical tissues or cultured astrocytes were extracted using TRIzol reagent (Invitrogen, USA) and quantified by NanoDrop (Thermo Fisher, USA). Subsequently, the reverse-transcribed cDNA was obtained PrimeScript™ II 1st Strand cDNA Synthesis Kit (Takara). Real-time PCR was performed using One Step TB Green® PrimeScript™ RT-PCR Kit (Takara) in an Applied Biosystems 7500 Real-Time PCR System using SYBR Green detection protocol as following conditions: 95 °C for 20 s, 40 cycles of 95°C for 5 s, and 60 °C for 30 s. The mRNA expression of GAPDH was set as the internal control. The results were expressed as fold changes compared with the sham group.

### Western blot analysis

Cells or tissues were extracted with cold RIPA buffer (1% Triton X-100, 0.1% SDS, 150 mM NaCl, 2 mM EDTA, 50 mM NaF, 10 mM sodium pyrophosphate, 1.0 mM Na3VO4, 1.0 mM PMSF, and complete protease inhibitor cocktail; Roche, Basel, Switzerland). The lysate was then centrifuged at 14,000 g for 15 min at 4 °C and the supernatant was collected for western blot analysis. The protein concentration of the samples was determined using a Pierce BCA Protein Assay Kit (Thermo Fisher Scientific, Waltham, MA, USA), and the tubes were stored at − 20°C. 10% SDS-PAGE was used to separate protein samples. After electrophoresis, the gels were transferred to polyvinylidene fluoride membranes (EMD Millipore, Burlington, MA, USA) using a constant current of 300 mV for 90 min. The membranes were then blocked in 5% milk in TBST (25 mM Tris-HCl, 150 mM NaCl, and 0.1% Tween 20, pH 7.4) for 30 min at room temperature on a rocker and incubated in the primary antibody overnight on a rocker at 4 °C. The membranes were washed with TBST and subsequently incubated with anti-rabbit or anti-mouse secondary antibodies for 1 h. The intensity of the protein signal was quantified with ImageJ software and normalized to the mean value of the sham group.

### Antibodies

For Western blot, the primary antibodies were as follows: rabbit anti AXL (1:500, bs-5180R, Bioss, Beijing, China), rabbit anti GAS6 (1:500, bs-7549R, Bioss), mouse anti GFAP (1:1000, MAB360, Millipore), rabbit anti CD68 (1:500, bs-0649R, Bioss), mouse anti ABCA1 (1:500, ab18180, Abcam, Cambridge, United Kingdom), rabbit anti IL-1β (1:500, ab9722, Abcam), rabbit anti TNFα (1:500, ab6671, Abcam), rabbit anti IL-6 (1:500, ab7737, Abcam), rabbit anti STAT1 (1:1000, 14994, CST), rabbit anti phospho-STAT1 (Tyr701) (1:1000, 9167, CST), and β-actin (1:1000, ab8227, Abcam). For immunofluorescence, the following antibodies were used: mouse anti GFAP (1:500, MAB360, Millipore), mouse anti NeuN (1:500, ab104224, Abcam), goat anti Iba-1 (1:500, ab5076, Abcam), rabbit anti AXL (1:500, bs-5180R, Bioss), rabbit anti GAS6 (1:500, bs-7549R, Bioss), and mouse anti ABCA1 (1:500, ab18180, Abcam). For flow cytometry, ACSA-2 antibody, anti-mouse, APC (1:50, 130-117-53, Miltenyi Biotec) and isotype control antibody, rat IgG2b, APC (1:50, 130-123-825, Miltenyi Biotec).

### Statistical analysis

Continuous data are presented as mean ± standard deviation (SD) or median (interquartile range) based on the normality and homogeneity of variance. Differences between two groups were analyzed using Student’s t-test or non-parametric test according to the normality and homogeneity of variance. The differences between three or more groups were analyzed by ANOVA or non-parametric tests. Categorical data are presented as n (%). Differences between groups were analyzed using the chi-square test, continuity correction, or Fisher’s exact test. All statistical analyses were performed using the SPSS 22 statistics software (SPSS Inc., Chicago, IL, USA). Statistical significance was indicated at p < 0.05.

## Result

### Microglia and astrocytes have specific reactive patterns in response to TBI

**Fig. 1:**
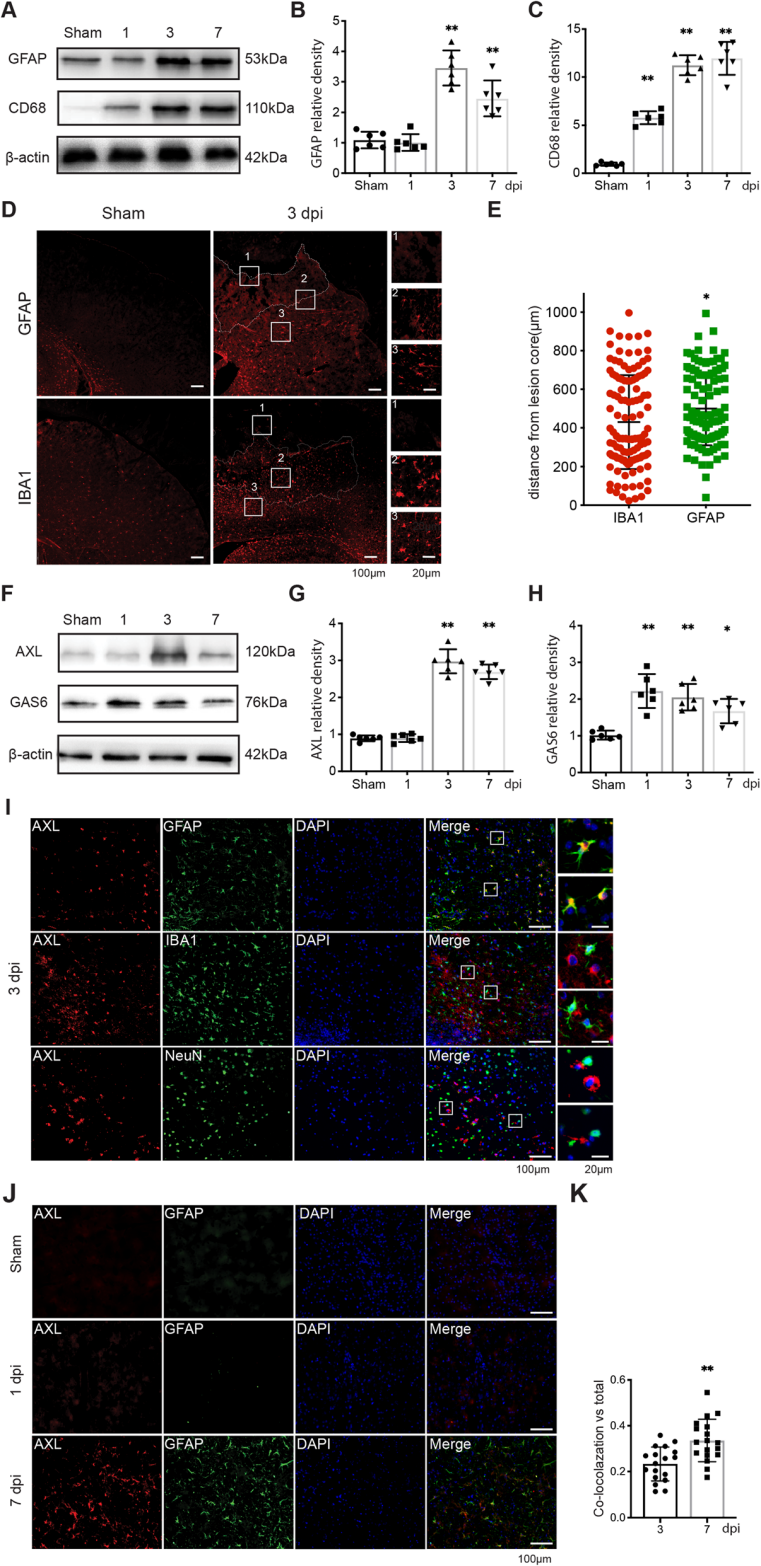
Time-dependent response of glial cells to traumatic brain injury. (**A**) Western blot of the time course of expression changes in GFAP and CD68. (**B**, **C**) The bar graphs show relative intensities for GFAP and CD68. The results are shown as the mean ± SD. **p < 0.01 by one-way ANOVA. (**D**) Reactive astrocytes surround damaged regions while microglia populate the center of the damaged region at 3 dpi. (**E**) Quantitative analysis for distances of IBA1 + activated microglia, and GFAP + reactive astrocytes from the center of the lesion in (**D**) from 4 mice. *p < 0.05 by two-tailed Student’s t-test. (**F**) Western blot samples reflect the changes in expression of AXL and GAS6 after TBI. (**G**, **H**) Quantitative analyses of AXL and GAS6 bands. *p < 0.05, **p < 0.01 by one-way ANOVA. (**I**) Immunofluorescence staining of GFAP, IBA1, NeuN (green), and AXL (red) in the ipsilateral cortex at 3 dpi; the nuclei were stained by DAPI (blue). (**J**) Immunofluorescence staining of GFAP (green), and AXL (red) at different time points post injury; the nuclei were stained by DAPI (blue). (**K**) Quantification analysis of the reactive astrocytes which expressed AXL kinase (GFAP+ & AXL+) after TBI in (**J**). n = 3 per group. **p < 0.05 by two-tail Student’s t-test

First, we established the TBI-induced activation patterns of microglia/macrophages and astrocytes. Western blotting showed that the expression of the glial fibrillary acidic protein (GFAP) in the ipsilateral side cortex was upregulated at 3 days post-injury (dpi) rather than 1 dpi (Fig. 1A, B). The expression of cluster of differentiation 68 (CD68), a marker of microglial activation was already upregulated at 1 dpi (Fig. 1A, C). Both GFAP and CD68 were upregulated at 7 dpi. Immunofluorescence showed GFAP+ signals were observed at 3 dpi in the injured side rather than contralateral, naive, or 1-dpi brains, and formed reactive astrocytes with prolonged processes and hypertrophic bodies (Supplemental Fig 1, Fig. 1D). Comparison with the distances between reactive astrocytes and microglia on the ipsilateral side at 3 dpi showed that these two types of glia had different spatial patterns in response to brain injury and the reactive astrocytes were surrounded the lesion core (Fig. 1E). Furthermore, WB analysis showed that the expression of AXL in the ipsilateral cortex was significantly increased at 3 dpi and 7 dpi, similar to GFAP expression (Fig. 1F, G). GAS6 had a similar pattern to CD68, which was highly increased early at 1dpi (Fig. 1F, H). Thus, to further determine whether AXL was located in astrocytes, we performed double immunofluorescence staining. At 3 dpi, AXL colocalized with GFAP+ reactive astrocytes around the damage core rather than with neuronal markers or microglia (Fig. 1I). Further analysis of the co-localized cells to total GFAP+ cell ratio showed that AXL was significantly upregulated in reactive astrocytes at 7 dpi compared with 3 dpi (Fig. 1I–K). Taken together, we identified the reactive pattern of two types of glia after brain injury and that AXL was upregulated in astrocytes as an adaptable response to TBI.

### The phagocytic capability of reactive astrocytes is mediated by AXL after TBI

**Fig. 2:**
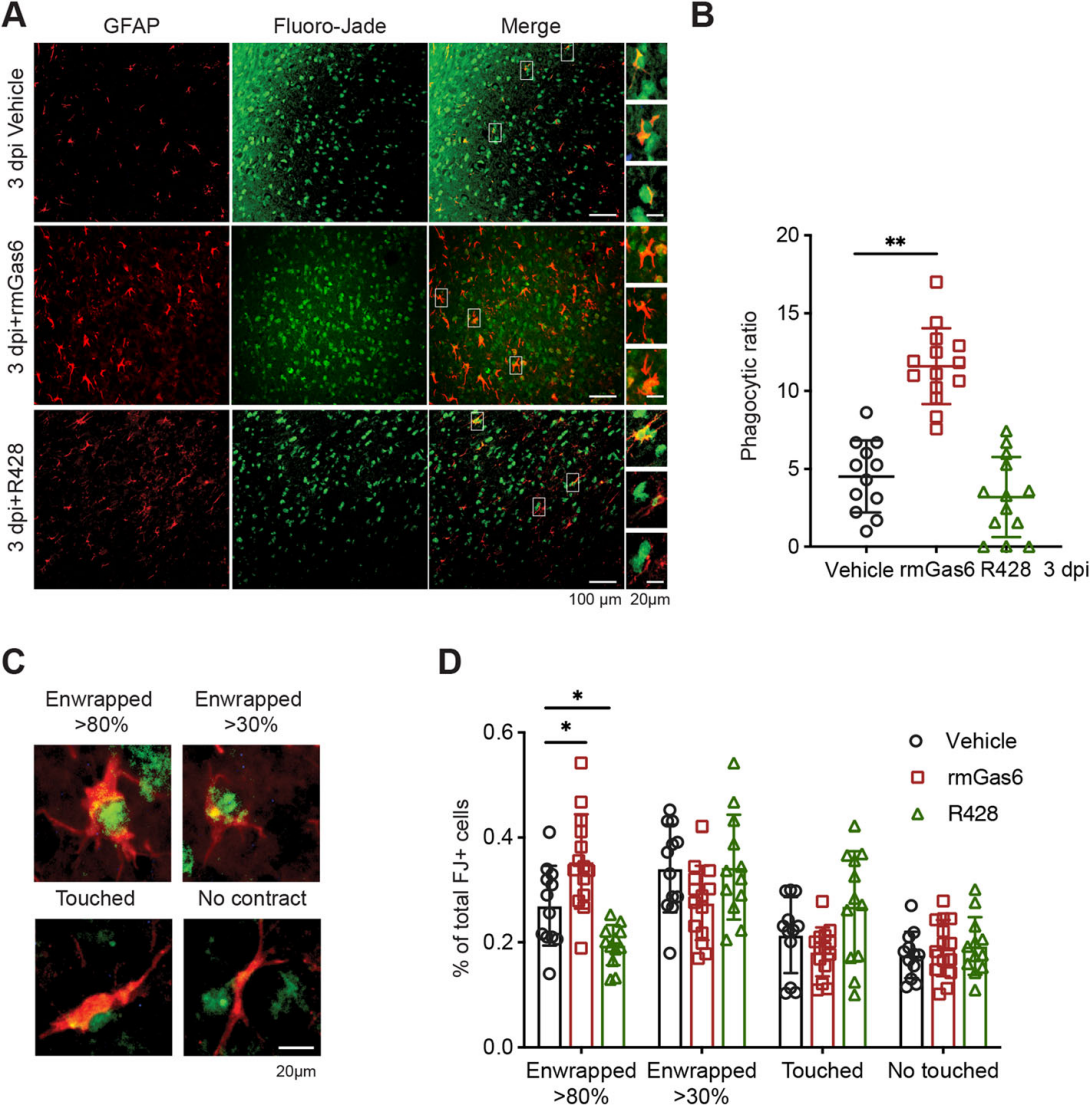
Astrocytes become phagocytes, mediated by AXL, after traumatic brain injury in vivo. (**A**) Representative images of Fluoro-Jade signals (green) and GFAP + (red) brain sections at 3 dpi with or without drug treatment and FJ + degenerating neuronal debris enwrapped by GFAP + astrocytes. (**B**) Quantification analysis of phagocytic GFAP+ astrocytes at 3 dpi following R428 or rmGas6 administration. **p < 0.01 by one-way ANOVA. (**C**) Images identified the degree of astrocytic phagocytosis (FJ+ neuronal debris enwrapped by GFAP+ astrocytes). (**D**) Quantification of four subtypes of phagocytic astrocytes. n = 4 per group. *p < 0.05 by two-way ANOVA

To assess whether AXL mediated the phagocytic features of transformed astrocytes after TBI, we measured the ingested FJ+ signals that marked degenerated neurons and neuronal debris in astrocytes as the index of astrocytic phagocytosis. At 3 dpi, FJ+ signals were enclosed by GFAP+ in the ipsilateral cortex, and astrocyte morphology was more conducive to capturing neurons or debris (Fig. 2A). Treatment with rmGas6 significantly increased the astrocytic phagocytosis ratio, while the AXL inhibitor abrogated the change without statistical difference (Fig 2A, B). Hence, according to previous work [16], we identified four degrees of astrocytic engulfment: enwrapped over 80%, enwrapped between 30 and 80%, contact, and no contact (Fig. 2C). Analyzing the proportion of each engulfment type and the proportion of total GFAP+ cells showed that after R428 treatment, the proportion of the enwrapped over the 80% subtype was significantly reduced, and rmGas6 administration enhanced this change (Fig. 2D). Taken together, this suggested that AXL participates in reactive astrocytic phagocytosis to remove damaged neurons or debris after TBI in vivo.

### AXL enhances the reactive astrocytic ingestion of neuronal debris

**Fig. 3:**
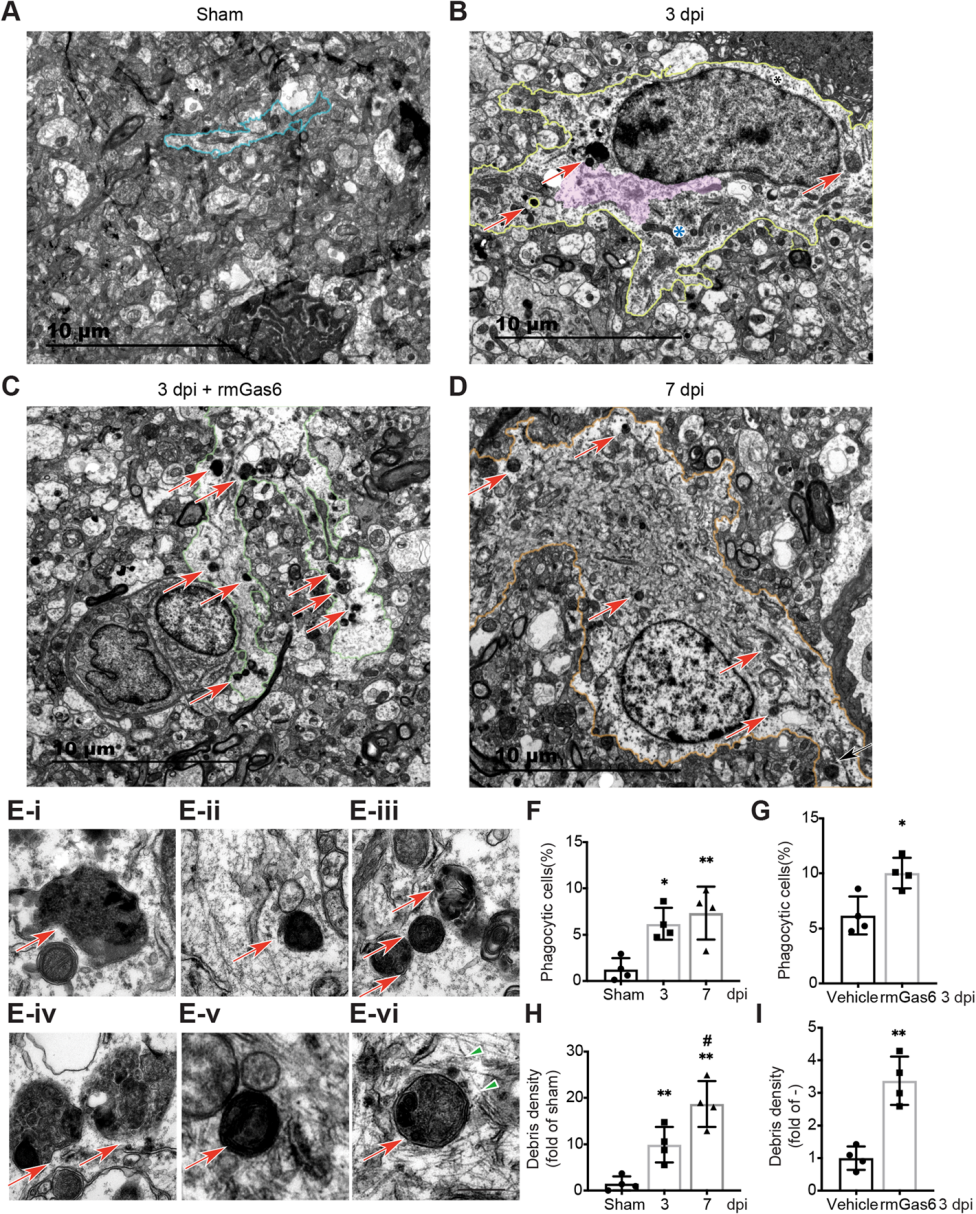
Phagocytosis of cellular debris in reactive astrocytes is mediated by AXL. (**A**–**D**) Representative TEM images of the astrocytes in the ipsilateral cortex (**A**, blue), those in the 3 dpi group were characterized by prominent intermediate filament bundles (**B**, pink area) and abundant glycogen granules (**B**, asterisks), the cytoplasm often contained small pieces of cellular debris (arrow). (**E**) The ingested debris was completely included in the astrocytic cytoplasm (arrow), and even situated in a cytoplasmic vacuole within astrocytes. (**F**) Analysis of phagocytic astrocytes participating in debris clearance after TBI. *p < 0.05, **p < 0.01 by one-way ANOVA). (**G**) rmGas6 increased the phagocytic astrocytes at 3 dpi. *p < 0.05 by two-tailed Student’s t-test. (**H**) Dot plots of debris density (number/area [μm^2^]) in astrocytes in the sham and 3 or 7 dpi ipsilateral cortex. **p < 0.01 versus sham, ^#^p < 0.05 versus 3 dpi by one-way ANOVA. (**I**) Astrocytic phagocytosis enhanced in rmGas6 treated ipsilateral cortex at 3 dpi. **p < 0.01 by two-tailed Student’s t-test

To validate the results of the immunofluorescence experiments, transmission electron microscopy (TEM) analysis was performed to detect phagocytic astrocytes after TBI. The astrocytes in the normal cortex (Fig. 3A) were thin and elongated, while those in the damaged cortex were characterized by prominent intermediate filament bundles (Fig. 3B pink area) and abundant glycogen granules (Fig. 3B asterisks) at 3 dpi. The cell bodies of reactive astrocytes had an extended cytoplasm which seems like a deformed pseudopod according to the astrocyte processes (Fig. 3A–D). Compared with the astrocytes of the sham group, the reactive ones included many cells or debris within the extended cytoplasm (Fig. 3A–D arrow). The same changes were observed at 7 dpi in the ipsilateral cortex (Fig. 3D) and rmGas6-injected mice at 3 dpi (Fig. 3C). After calculating the ratio of phagocytic astrocytes and the debris density in these reactive astrocytes, we observed that the phagocytosis capability of astrocytes remarkably increased at 3 and 7 dpi compared with the sham group (Fig. 3F, H). Furthermore, rmGas6 promoted the reactivity of astrocytes, which were transformed to phagocytic cells at 3 dpi (Fig. 3G, I). The engulfed cells or debris in the cytoplasm displayed hallmark chromatin traits of apoptosis (Fig. 3E-i–iii), and a ruptured cell membrane, signified as subsequent necrosis (Fig. 3E-iv–v). The engulfed cells or debris appeared to be contained in vacuolar structures with surrounding actin filaments (Fig. 3E-vi, arrowhead). Thus, the astrocytes transformed into cells with characteristics of phagocytes after TBI, and rmGas6, the agonist of AXL, promoted the transformation.

### AXL mediates astrocytic phagocytosis triggering apoptosis cells in vitro

**Fig. 4:**
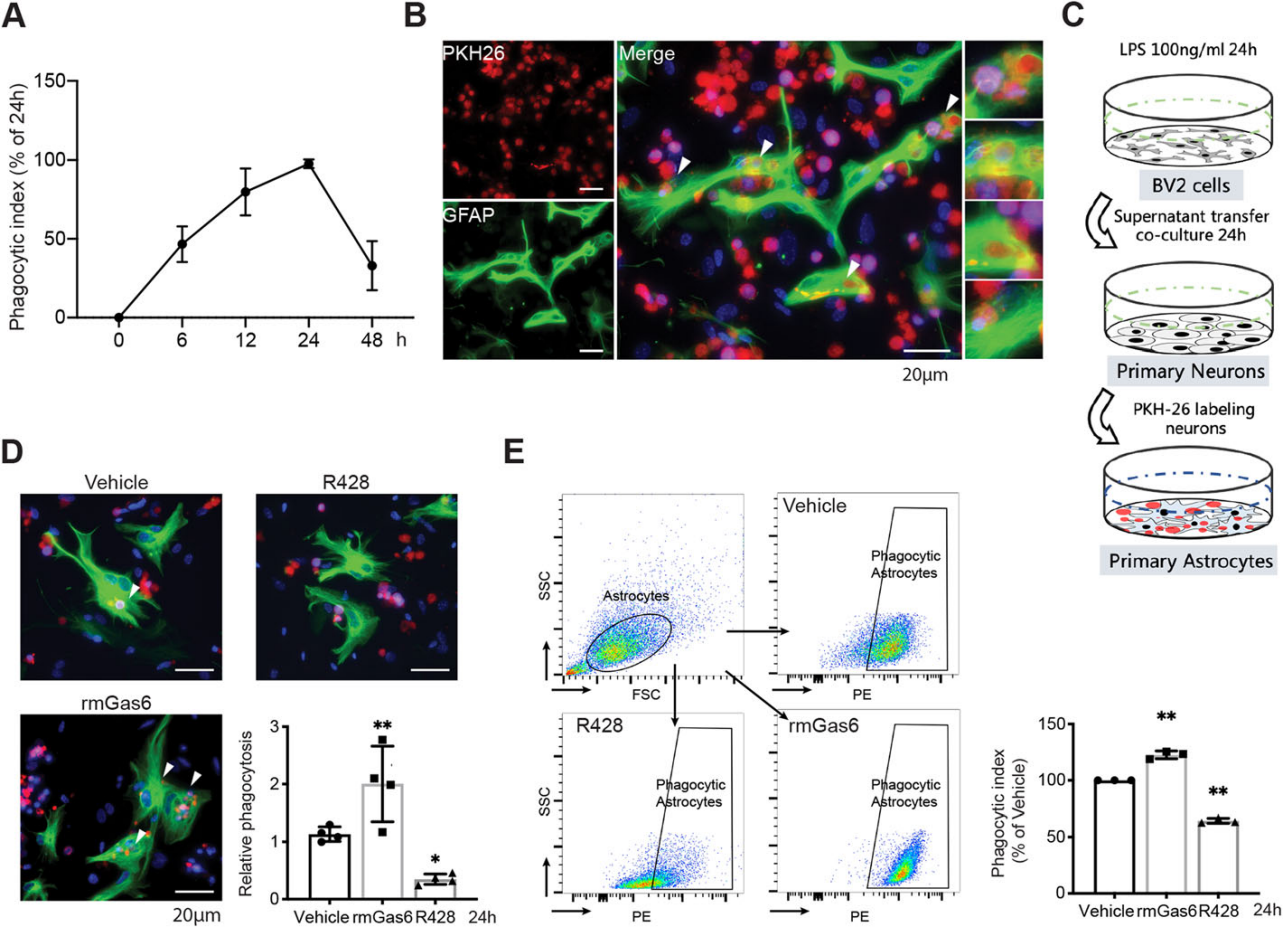
AXL mediates the phagocytic transformation of astrocytes in vitro. (**A**) Flow cytometry indicated the temporal phagocytosis ratio change of astrocytes. n = 3 per time point. (**B**) Representative images of PKH26 labeled apoptotic neurons (red) engulfed by astrocytes (green). The phagocytic astrocytes had a phagocytic cup structure (arrowhead) that contained PKH26+ debris or cells. (**C**) Brief process of in-vitro astrocytic phagocytosis analysis. (**D**) Immunofluorescence staining of astrocytic phagocytosis and quantification analysis of phagocytosis ratio with rmGas6 or R428 treatment after 24 h co-incubated. n = 3 per group. *p < 0.05, **p < 0.01 by one-way ANOVA. (**E**) The astrocytic ingestion was assessed by flow cytometry after 24 h co-culture. Astrocytes were pretreated with R428 or rmGas6 for 1 h prior to the addition of PKH+ neurons. n = 3 per group. **p < 0.01 by one-way ANOVA

To further demonstrate the role of AXL in astrocytic phagocytosis, we set up a cell model: primary astrocytes were incubated with insulted primary neurons pretreated with cell-free supernatant from LPS-stimulated microglia for 24 h (Fig. 4C). We detected phagocytic ratio over time after co-culture with PKH26-labeled neurons by flow cytometry, and the results showed that astrocytic engulfment peaked at 24 h in vitro (Fig. 4A). Moreover, immunofluorescence indicated that astrocytes formed a phagocytic cup structure (Supplemental Fig 3), a special alteration of astrocytic cytoplasm that contains ingested cells or debris, after 24 h of co-culture (Fig. 4B). Astrocytic phagocytosis was significantly decreased after exposure to R428 while rmGas6 induced the opposite effects: the count of ingested neurons in astrocytes was significantly increased (Fig. 4D). These results were further confirmed by flow cytometry analysis; regardless of the drug-treated or siRNA-treated cell model, the astrocytic phagocytosis was partly dependent on AXL (Fig. 4E, Supplemental Figure 4). Together, these findings suggested that astrocytes could acquire phagocytic capability to remove damaged neurons or debris, and this is mediated by AXL in vitro.

### AXL upregulates the phagocytosis-associated molecule ABCA1 via STAT1 in astrocytes is essential for neuroprotection

**Fig. 5:**
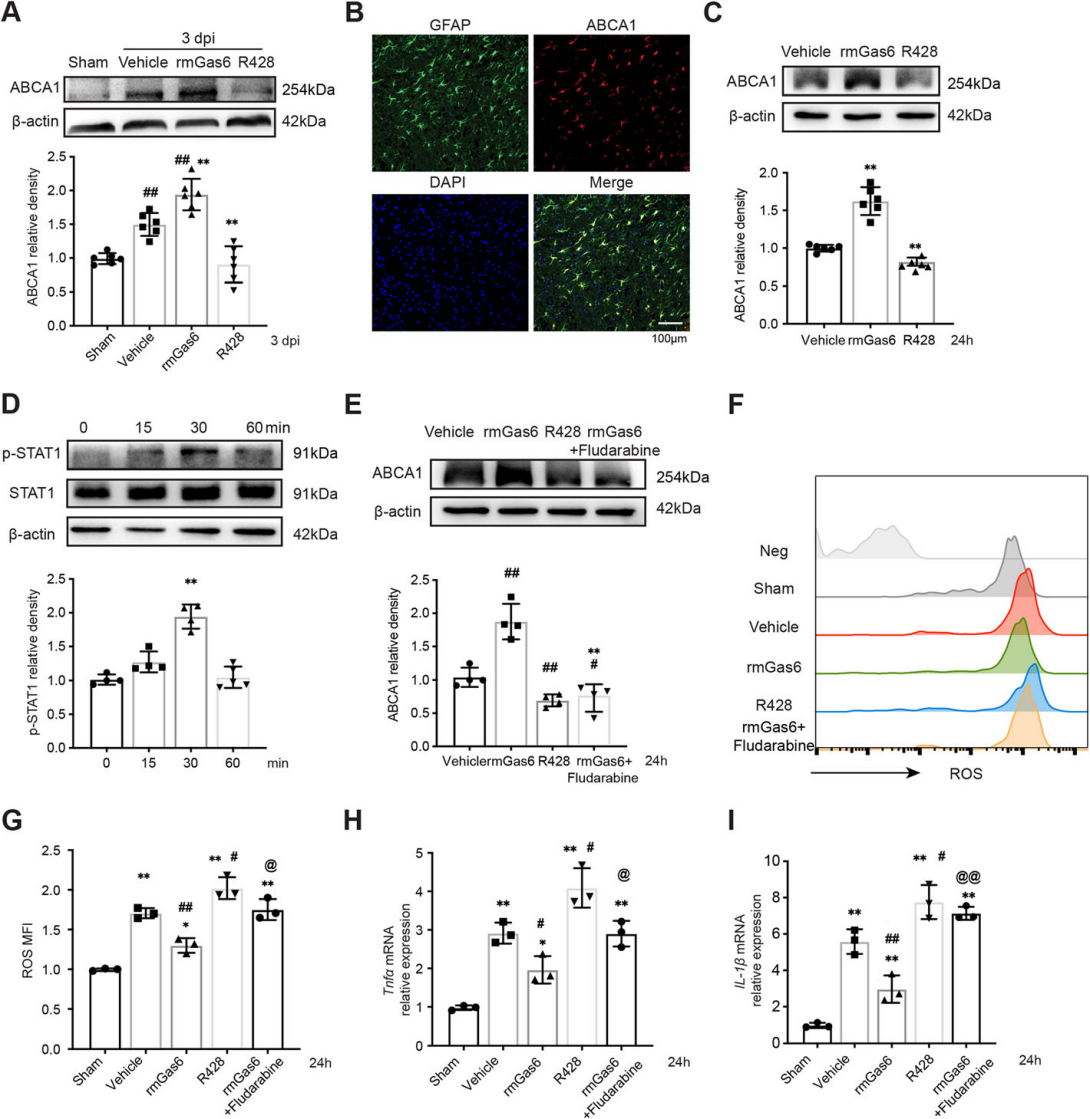
ABCA1 is a phagocytosis-associated molecule mediated by AXL in astrocytes. (**A**) Expression of ABCA1 is detected after AXL intervention at 3 dpi. **p<0.01 versus sham, ^##^p<0.01 versus vehicle by one-way ANOVA. (**B**) Immunofluorescence staining of ABCA1 (red) and GFAP (green) colocalization at 3 dpi in the ipsilateral cortex after rmGas6 treatment. (**C**) Immunoblot of ABCA1 in primary astrocytes with or without drug administration. *p < 0.05, **p < 0.01 by one-way ANOVA. (**D**) Representative Western blot images and quantitative analyses of p-STAT1/STAT1 in primary cultured astrocytes stimulated by rmGas6 at different time points. **p < 0.01 versus 0 min by one-way ANOVA. (**E**) Representative Western blot images and quantitative analyses of ABCA1 in primary astrocytes. **p < 0.01 versus vehicle, ^##^p < 0.01 versus rmGas6 by one-way ANOVA. (**F**) Representative histograms show the ROS level in astrocyte samples from each group. (**G**) Quantification of percentage of ROS expression in astrocytes. *p < 0.05, **p < 0.01 versus sham, ^#^p < 0.05, ^##^p < 0.01 versus vehicle, @p<0.05 versus rmGas6 by one-way ANOVA. (**H**, **I**) Relative TNFα and IL-1β mRNA expression in astrocytes from each group. **p < 0.01 versus sham, ^#^p < 0.05, ^##^p < 0.01 versus vehicle, ^@^p < 0.05, ^@@^p < 0.01 versus rmGas6 by one-way ANOVA

Previous studies have reported that ABCA1 mRNA and protein are both significantly increased after TBI [36] and that ABCA1 is associated with astrocytic phagocytosis in ischemic injury [16]. Hence, we evaluated the impact of AXL intervention on ABCA1 expression in astrocytes. At 3 dpi, the ABCA1 protein level was significantly upregulated; rmGas6 enhanced the upregulation while R428 blocked this change (Fig. 5A). Co-localization analysis by immunofluorescence suggested that ABCA1 was highly expressed in astrocytes after rmGas6 treatment at 3 dpi (Fig. 5B). In primary cultured astrocytes, we also confirmed the association between AXL and ABCA1. In the peak stage of astrocytic phagocytosis (24 h), ABCA1 levels were significantly increased after rmGas6 stimulation, whereas R428 administration significantly downregulated its expression (Fig. 5C). We noticed that the signaling transduction of ABCA1 induced by LXR transcription of TAM receptors related with STAT1 participation in macrophages [37]. Thus, we wondered whether STAT1 modified AXL-induced astrocytic phagocytosis. In primary cultured astrocytes, we detected the time course of STAT1 phosphorylation (tyrosine 701) after rmGas6 stimulation. The results indicated phospho-STAT1 (tyrosine 701) rapidly peaked within 30 min and declined to the baseline at 60 min post stimulation (Fig. 5D). Further, we introduced a selective inhibitor of STAT1: fludarabine and pretreated before rmGas6 administration; the expression of the ABCA1 protein was detected from harvested cells at 24 h. The results showed rmGas6 upregulated the expression of ABCA1 while R428 inhibited the expression. In addition, fludarabine blocked the rmGas6-induced upregulation of ABCA1 in astrocytes (Fig. 5E). As reactive oxygen species (ROS) and inflammatory cytokines generated by astrocytes have been considered to play critical roles in astrocyte-mediated neuronal injury, we next measured the ROS and inflammation levels in each group of the in vitro model. In the primary cultured astrocytes, we detected less ROS in the rmGas6-treated group compared with the vehicle group but the protective effect of rmGas6 was abolished by STAT1 inhibitor, and R428 notedly promoted the production of ROS (Fig. 5F, G). R428 also induced the expression of inflammatory genes like TNFα and IL-1β, and the suppression of inflammatory transcription induced by rmGas6 was remarkably blocked by fludarabine treatment (Fig. 5H, I). These data suggested that AXL increased the expression of another phagocytosis molecule ABCA1, through STAT1 phosphorylation in reactive astrocytes which contributed to astrocyte phagocytosis and neuroprotection.

### AXL attenuates TBI induced neuroinflammation and behavior deficits

**Fig. 6:**
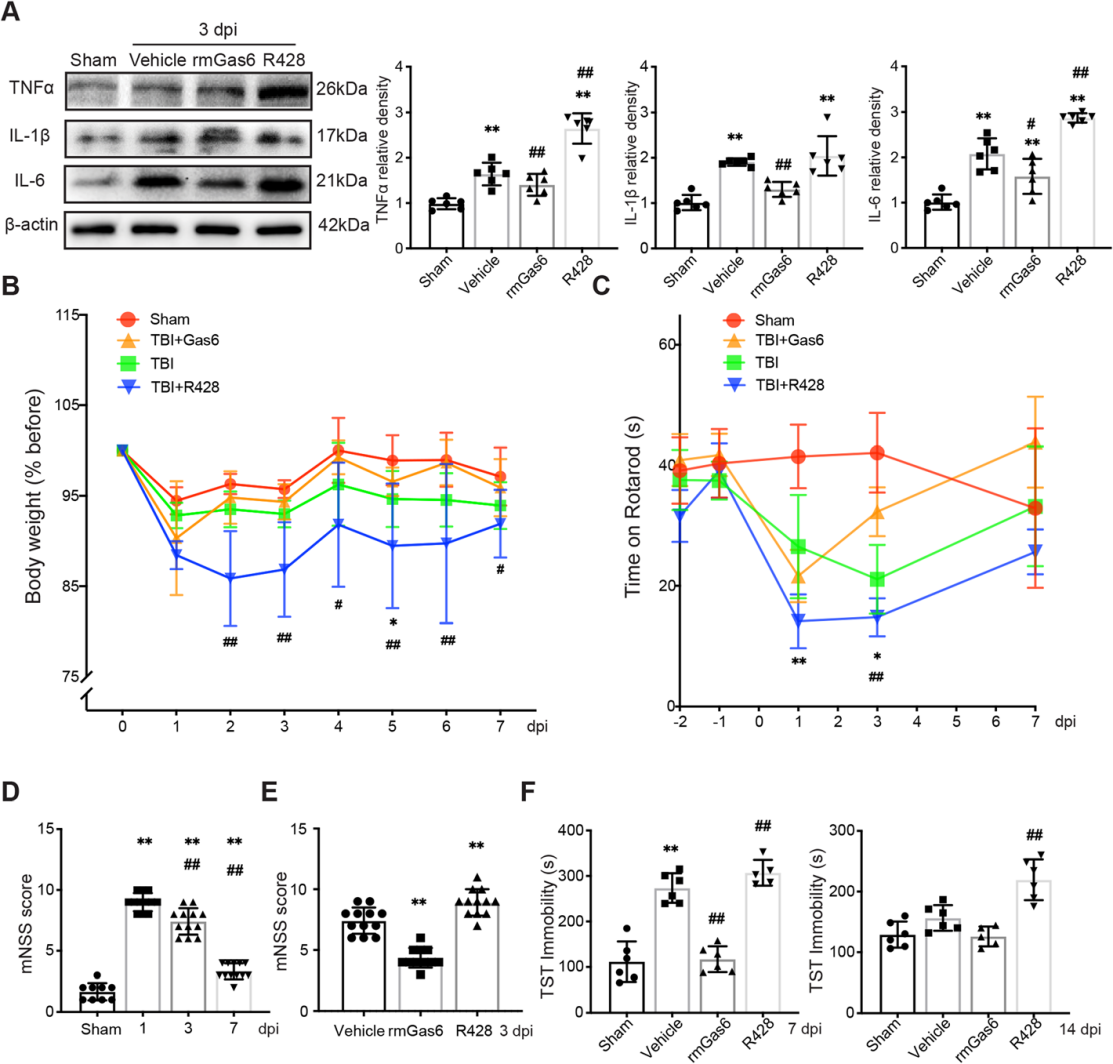
AXL influences TBI-induced neuroinflammation and neurological impairments. (**A**) Inflammation cytokines are determined at 3 dpi under drug treatment. IL-6/TNFα/IL-1β were upregulated at 3 dpi and rmGas6 treatment attenuated the inflammation upregulation, whereas R428 treatment intensified neuroinflammation. **p < 0.01 versus sham, ^#^p < 0.05, ^##^p < 0.01 versus vehicle by one-way ANOVA. (**B**) Drug treatment mediates TBI-induced body weight changes. The relative change of body weight was calculated by comparing with 0 dpi. *p < 0.01 versus sham, ^#^p < 0.05, ^##^p < 0.01 versus vehicle group by two-way ANOVA. (**C**) Motor coordination was reduced by TBI, and the disorder was enhanced by R428 treatment while rmGas6 attenuated this change. **p < 0.01, *p < 0.05 versus sham, ^##^p < 0.01 versus vehicle by two-way ANOVA. (**D**) Temporal neurological function assessment of mice suffered TBI at different time points by using modified neurological severity score test (mNSS). **p < 0.01 versus sham, ^##^p < 0.01 versus 1 dpi by one-way ANOVA. (**E**) mNSS at 3 dpi under drug treatment. **p < 0.01 by one-way ANOVA. (**F**) Depressive-like behavior was determined after TBI with/ without drug administration by time spent immobile in the TST at 7 dpi (left) and 14 dpi (right). All data are presented as mean ± SD; (n = 6). **p < 0.01 versus sham, ^##^p < 0.01 versus vehicle by one-way ANOVA

To determine whether the AXL-induced transformation of reactive astrocytes is associated with the outcome of TBI, we evaluated the neurological functional changes after trauma. It is well known that neuroinflammation is the main pathological process post injury caused by damaged or necrotic tissues. Western blot analysis of the ipsilateral cortex showed that the inflammation factors IL-6, TNFα, and IL-1β were significantly upregulated at 3 dpi, while rmGas6 treatment caused inflammation remission. At the same time, R428 caused severe production of inflammatory factors such as TNFα and IL-6 (Fig. 6A). The analysis of body weight change showed that R428 intensified TBI-induced weight loss at 2–7 dpi compared with the TBI group, while rmGas6 had the converse effect (Fig. 6B). The rotarod test showed that TBI mice had impaired motor coordination compared to baseline and sham levels. rmGas6 attenuated motor impairment at 3 dpi, while R428 increased motor deficit early at 1 dpi (Fig. 6C). The results of the mNSS score suggested that neurological recovery returned to baseline at 7 dpi; thus, we chose 3 dpi for further research (Fig. 6D). We found that rmGas6 improved neurological function in mice at 3 dpi, in contrast to R428, which exacerbated the behavioral deficits (Fig. 6E). Depression is one of the complications after TBI related to neuroinflammation in the long phase [38]. Thus, we tested TBI mice for behavioral resignation determined by immobility in the TST at 7 dpi, the time when all groups had comparable weights and motor coordination. The results showed that TBI mice spent more time immobile in the TST compared to the sham group, and rmGas6-treated mice spent less time immobile compared to the TBI vehicle group. At 14 dpi, the R428-treated mice spent more time immobile compared to the TBI vehicle group (Fig. 6F). Taken together, AXL-induced astrocytic transformation could attenuate neuroinflammation and neurological behavior impairments after TBI.

### Microglia influence astrocytic phagocytosis via derived GAS6 after TBI in vivo and in vitro

**Fig. 7:**
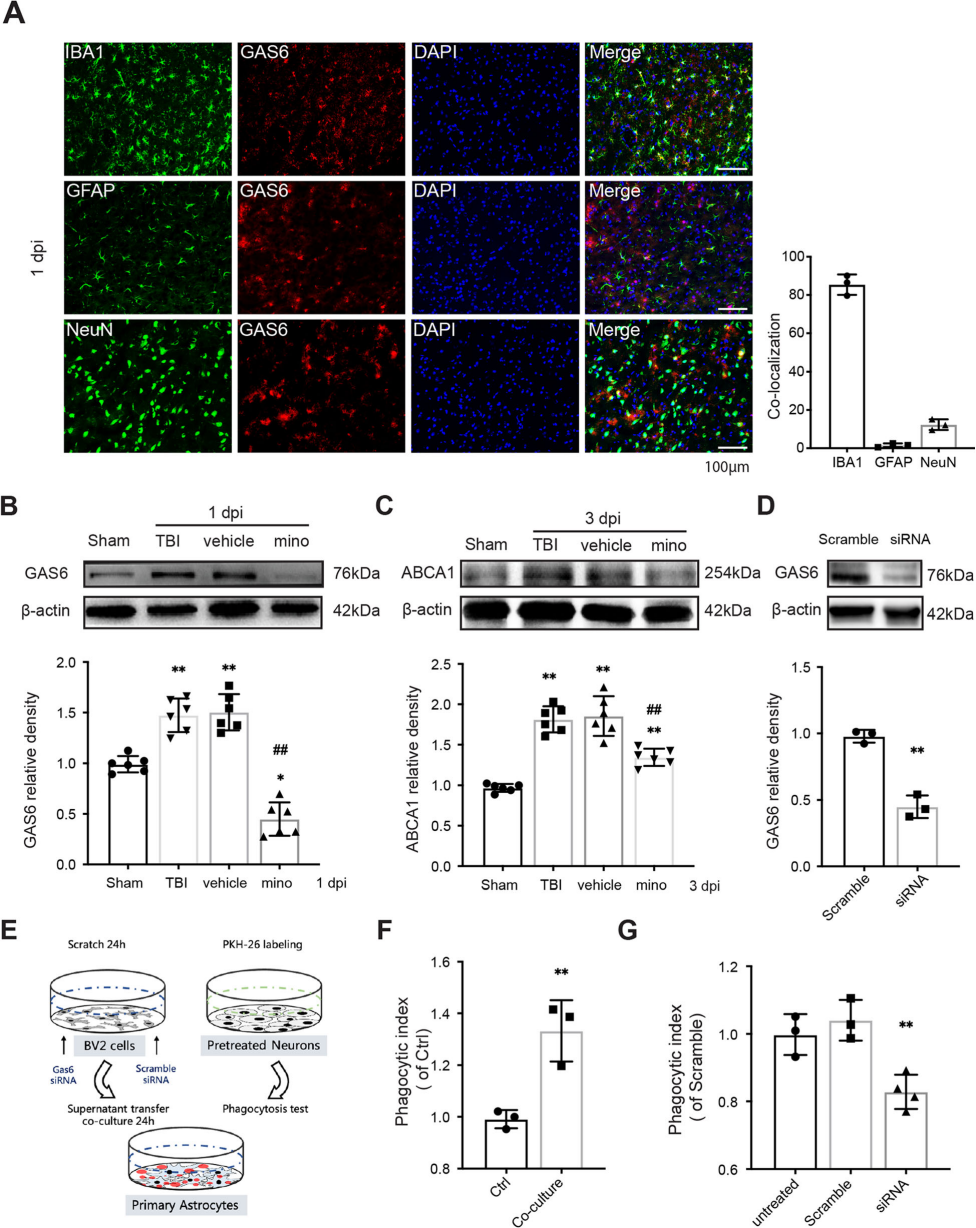
Microglia influence astrocytic transformation via the GAS6/AXL axis. (**A**) Immunofluorescence staining of GAS6 (red) with IBA1, GFAP, or NeuN (green) after TBI; nuclei were stained by DAPI (blue). (**B**) GAS6 level was suppressed by minocycline administration at 3 dpi. *p < 0.05 **p < 0.01 versus sham, ^##^p < 0.01 versus vehicle by one-way ANOVA. (**C**) ABCA1 level was suppressed after minocycline treatment at 3 dpi. *p < 0.05, **p < 0.01 versus sham, ^##^p < 0.01 versus vehicle by one-way ANOVA. (**D**) The effect of GAS6 specific targeted siRNA or scramble siRNA after transfected into BV2 cells. *p < 0.05 by two-tailed Student’s t-test. (**E**) Brief workflow of in-vitro cell crosstalk analysis. (**F**) BV2 supernatant enhanced primary astrocyte phagocytosis after 24 h co-culture, as detected by flow cytometry. **p < 0.01 by two-tailed Student’s t-test. (**G**) Flow cytometry analysis of BV2 supernatant-induced astrocytic phagocytosis showed it was abolished after GAS6 siRNA transfection. **p < 0.01 versus scramble by one-way ANOVA

As GAS6 has been reported to be transcriptionally upregulated in microglia after TBI [39], we hypothesized that AXL-associated astrocytic phagocytosis may be initiated by microglia-derived GAS6. Our results confirmed that GAS6 protein was mainly colocalized with IBA-1+ microglia (over 80%) at 1 dpi (Fig. 7A). Next, we selectively inhibited the activation of microglia using minocycline (50 mg/kg intraperitoneally) [4]. Minocycline treatment significantly inhibited microglia activation and downregulated GAS6 levels at 1 dpi and suppressed the expression of ABCA1 at 3dpi (Fig. 7B, C). In the in vitro model of microglia-astrocyte coordination (Fig. 7E), our data suggested that the BV2 cell-free supernatant could promote astrocytic ingestion of PKH26-labeled, pretreated neurons, as detected by flow cytometry (Fig. 7F). The GAS6-deficient supernatant prepared by selective siRNA was transfected into BV2 cells (Fig. 7D). The GAS6-deficient supernatant significantly abrogated the effect of promoting astrocytic phagocytosis compared with the supernatant from scramble siRNA-transfected BV2 cells while the scramble siRNA-treated BV2-derived supernatant has a similar effect with the untreated BV2 supernatant (Fig. 7G). These results suggested that microglia can regulate the astrocytic transformation and phagocytosis which is mediated by AXL signaling transduction via the secretion of GAS6.

## Discussion

**Fig. 8:**
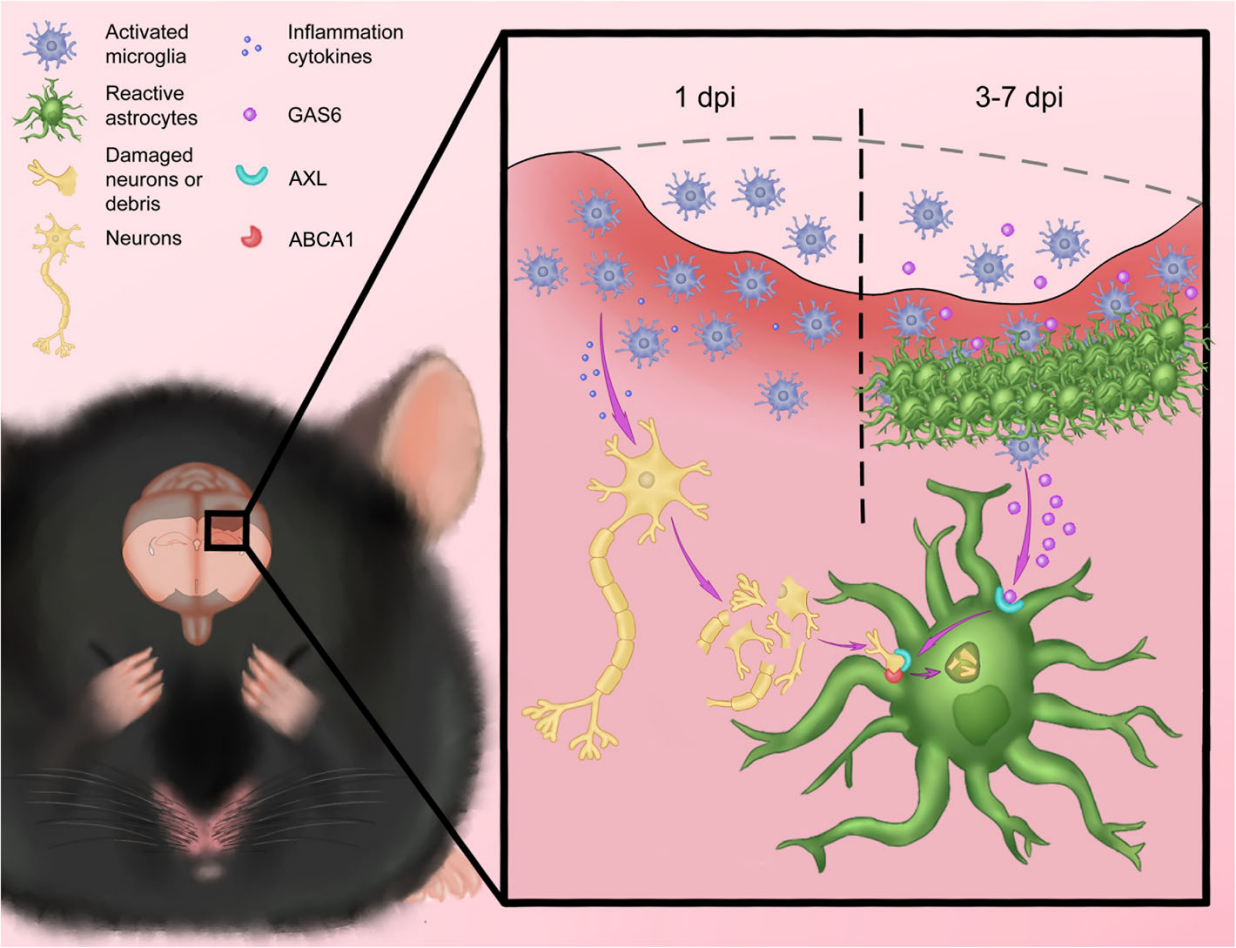
The schematic diagram demonstrating that AXL which initially activated by microglia derived GAS6 modulates transformation of astrocytes from resting to phagocytic phenotype through STAT1-ABCA1 signaling pathway to alleviate the inflammation and ROS injury after TBI

In the current study, we demonstrated the existence of astrocytic transformation to phagocytes after TBI and showed that AXL plays an indispensable role in this transformation as a novel target. Engulfed apoptotic cells or fractions of degenerating neuronal cell debris were observed in reactive astrocytes in the peri-injured region. This may act as a “defensive wall,” preventing bystander killing and cytotoxic factors from damaging normal tissue (Fig. 8).

Astrocytes actively clip synapses by phagocytosis in the lateral geniculate nucleus during brain development and are reserved in the adult brain, although the efficacy of the adult state is much lower [40]. After acute brain injury, the cerebral microenvironment undergoes dynamic structural and functional changes that share a similarity with the developmental neuronal system [41], suggesting that under these conditions, the brain or neurons are more plastic. Both in adults and during development, naturally or pathologically occurring bystander killing has been identified as a type of neuron death, such as in the retina neurons [42]. An alternative explanation of this might result from contact between primary cells which have undergone necroptosis or apoptosis and healthy cells with death receptors expressed on, including Fas and tumor necrosis factor receptor-1 [43]. In contrast to neurons, astrocytes have been shown to be resistant to apoptosis-induced bystander killing via the Fas-ligand [44], thus reminding us of the potential to limit the bystander effect via glia. Phagocytes rapidly remove unhealthy cells and debris from damaged tissue to prevent the uncontrolled release of cytokines and inflammation, which has been shown to have a beneficial effect on prolonged social withdrawal, resignation, and anhedonia [45]. Our data showed that in response to TBI, astrocytes changed to a phenotype with prolonged processes and hypertrophic bodies at 3 and 7 dpi (Fig. 2). TEM detected phagosomes that contained engulfed cells or debris in the cytoplasm of astrocytes within the damaged cortex, in contrast to the normal astrocytes in the healthy cortex. Thus, we determined that the phagocytic transformation of astrocytes is not a physiological event, but rather occurs in pathophysiological conditions as a potential protection mechanism. Our further studies both in vivo and in vitro were in accordance with this hypothesis. We define these reactivate astrocytes as a “defensive wall” according to their spatiotemporal patterns and attribute the phagocytic feature to limiting damaged tissue to reduce the secondary injury from bystander killing and neuroinflammation. In this case, we do not consider astrocytic phagocytosis as the only or even the primary way that degenerated neurons are removed after TBI. We suppose that phagocytic astrocytes support microglia in debris clearance, while microglia mainly work in the core damage region via the rapid response. To better understand astrocytic phagocytosis in response to damage, further studies are needed to identify the phagocytosis characteristics of astrocytes and microglia, such as the size of ingestion, lysosome activity, and cell metabolism.

The phagocytosis of apoptotic cells requires recognition of phosphatidylserine (PtdSer) on the membrane [46, 47]. The TAM family of receptors are well-known PtdSer receptors that have been demonstrated to play a role in phagocytosis in retinal pigment epithelial cells, Sertoli cells in the testis, Schwann cells in peripheral nerves, and astrocytes in the brain [14, 48]. In the present study, we provide new evidence of the role of AXL in the injured brain, where it mediates astrocytic phagocytosis. We showed that the temporal upregulation of AXL was similar to that of GFAP after TBI and reactive astrocytes were colocalized with strong AXL expression (Fig. 1). AXL altered astrocytic phagocytosis both in vitro and in vivo (Figs. 2, 3 and 4) and influenced TBI-induced neurological impairments (Fig. 6). We also revealed a mechanism about cellular crosstalk between microglia and astrocytes as early activated microglia produced the GAS6-modulated astrocyte phenotype and phagocytosis function both in and ex vivo (Fig. 7). Thus, our work builds on previous studies innovatively showing an overall operation between the types of glia via the GAS6-AXL axis during disease progression. Further, it is noteworthy that ABCA1 has been identified as a regulator of astrocytic phagocytosis in cerebral ischemia [16]. ABCA1 activity is known to have anti-inflammatory and ROS-reducing effects in macrophages [49], but its activity is not clear in astrocyte inflammation and ROS production. Based on our work, AXL might work synergistically with ABCA1 in astrocytic phagocytosis (Fig. 5) as rmGAS6 rapidly enhanced STAT1 phosphorylation at Tyr-701 in astrocytes in a similar time course consistent with previous reports [37]. In addition, AXL could reduce the neuroinflammation and ROS level in astrocytes which were recognized as significant characters of unhealthy astrocytes after brain injury (Figs. 5 and 6). In professional phagocytes, the promotion of phagocytosis is beneficial to limit inflammation and injury recovery through autophagy [50], metabolic reprogramming [20, 51], and ant-inflammatory cytokine release [51]. Our work demonstrates that the AXL-mediated transformation of astrocytes remarkably enhanced the phagocytic capability and efficaciously against TBI-induced damage. Further studies are needed to provide a better understanding of the physiological consequences of astrocytic phagocytosis and to investigate the other potential mechanism of phagocytic modulation, such as autophagy, energy metabolism.

Approximately 30–40% of neurological impairments and depressive symptoms that occur in human TBI patients are associated with glial overbalance and inflammation [52, 53]. In our study, we found that TBI mice spent less time on the rotarod immediately after TBI, but this returned to baseline within 7 days, while depressive-like behavior was detected at 7 and 14 days when each group showed equal motor capability and body weight. Moreover, TBI-induced motor disorders and depressive behavior were related to drug administration, which means these disorders may point to AXL-mediated astrocytic transformation (Fig. 6). Thus, based on AXL-induced phagocytic astrocytes that respond to active microglia-derived GAS6 after TBI, we connected the glial function with acute neurological and later psychiatric disorders that could be manipulated in the early stages.

In conclusion, the present study establishes a mechanism by which the transformation of astrocytes to a phagocytic phenotype is initially activated by microglia through the GAS6/AXL pathway, contributing to separate healthy brain tissue from injury-induced cell debris, and ameliorating neurological impairments after TBI. This novel pathophysiological mechanism is a potential target for refining therapeutic approaches.

## Conclusions

Our works set up a mechanism, the transformation of a phagocytic phenotype in astrocytes is initially activated by microglia through the GAS6/AXL pathway, contribute to separate healthy brain tissue from injury-induced cell debris, and then ameliorate neurological/psychiatric impairments after TBI. Furthermore, pharmacological activation of AXL could attenuate inflammatory injury via the AXL/STAT1/ABCA1 pathway-induced phagocytic transformation of astrocytes. Thus, the current study supports the notion that targeting AXL might be a novel therapeutic strategy for TBI.

## Supplementary Information

**Additional file 1:.** Supplementary Figure 1. Immunofluorescence staining of GFAP at 3 days post TBI, the reactive astrocytes were detected in the ipsilateral section instead of the contralateral area. Scale bar = 10μm

**Additional file 2:.** Supplementary Figure 2. The purity of primary cultured astrocytes. APC-ACSA-2 was used for detecting the percentage of astrocytes in the cultured cells by flow cytometry

**Additional file 3:.** Supplementary Figure 3. Representative images of phagocytic cup during astrocytic phagocytosis. The engulfed PKH26 labeled neuron (red) was surrounding by abundant F-actin (arrowhead) which is visualized by Phalloidin (green). Scale bar = 10μm

**Additional file 4:.** Supplementary Figure 4. The astrocytic phagocytosis was assessed by flow cytometry after 24 h co-culture. Astrocytes were pretreated with AXL-siRNA or scramble siRNA for 48h prior to the addition of PKH26 labeled neurons. n=3 per group. **p<0.01 by two-tail Student’s t-test

**Additional file 5:.** Supplementary Information: text summary

**Additional file 6:.** Supplementary table 1. mNSS protocols

## Data Availability

All raw data used in this manuscript are available on reasonable request.

## Abbreviations

TBI: Traumatic brain injury
AXL: AXL receptor tyrosine kinase
STAT1: Signal transducer and activator of transcription 1
GAS6: Growth arrest specific 6
rmGas6: Recombinant mouse Gas6 protein
CCI: Controlled cortical impact
RT-PCR: Real-time polymerase chain reaction
GFAP: Glial fibrillary acidic protein
CD68: Cluster of differentiation 68
dpi: Day post-injury
WB: Western Blot
FJ: Fluoro-Jade C
ABCA1: ATP-binding cassette transporter 1
mNSS: Neurological severity scores
TST: The tail suspension test
PBS: Phosphate-buffered saline
PFA: paraformaldehyde
DMSO: Dimethyl sulfoxide
ROS: Reactive oxygen species
PDL: Poly-d-lysine
