# Supplementary figures and images for: AXL kinase-mediated astrocytic phagocytosis modulates outcomes of traumatic brain injury
Source: J Neuroinflammation. 2021 Jul 7;18:154. doi: 10.1186/s12974-021-02201-3 (PMC8264993; doi:10.1186/s12974-021-02201-3)

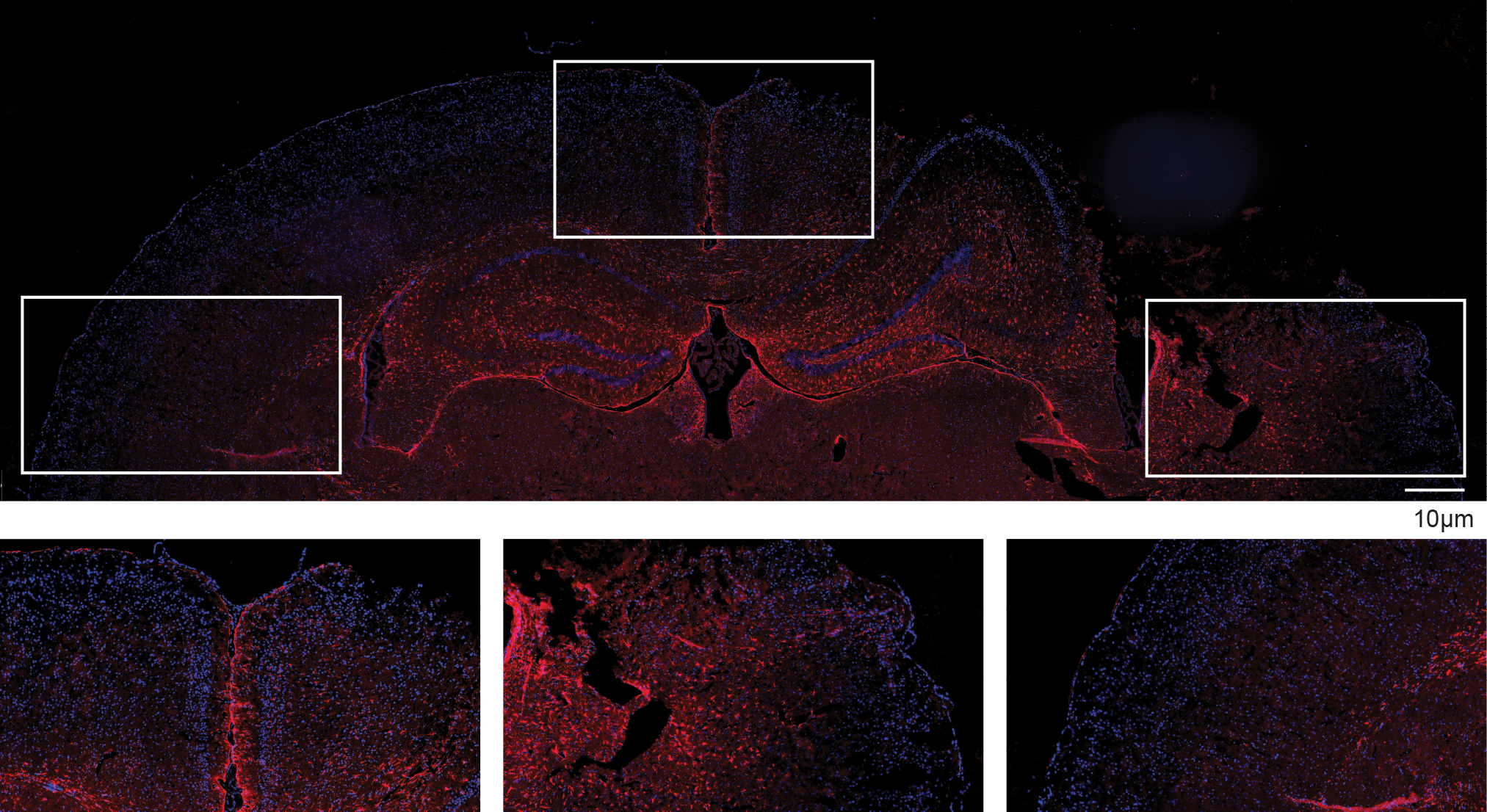

Supplement: Supplementary file 1 — Additional file 1:. Supplementary Figure 1. Immunofluorescence staining of GFAP at 3 days post TBI, the reactive astrocytes were detected in the ipsilateral section instead of the contralateral area. Scale bar = 10μm [file 12974_2021_2201_MOESM1_ESM.tif]

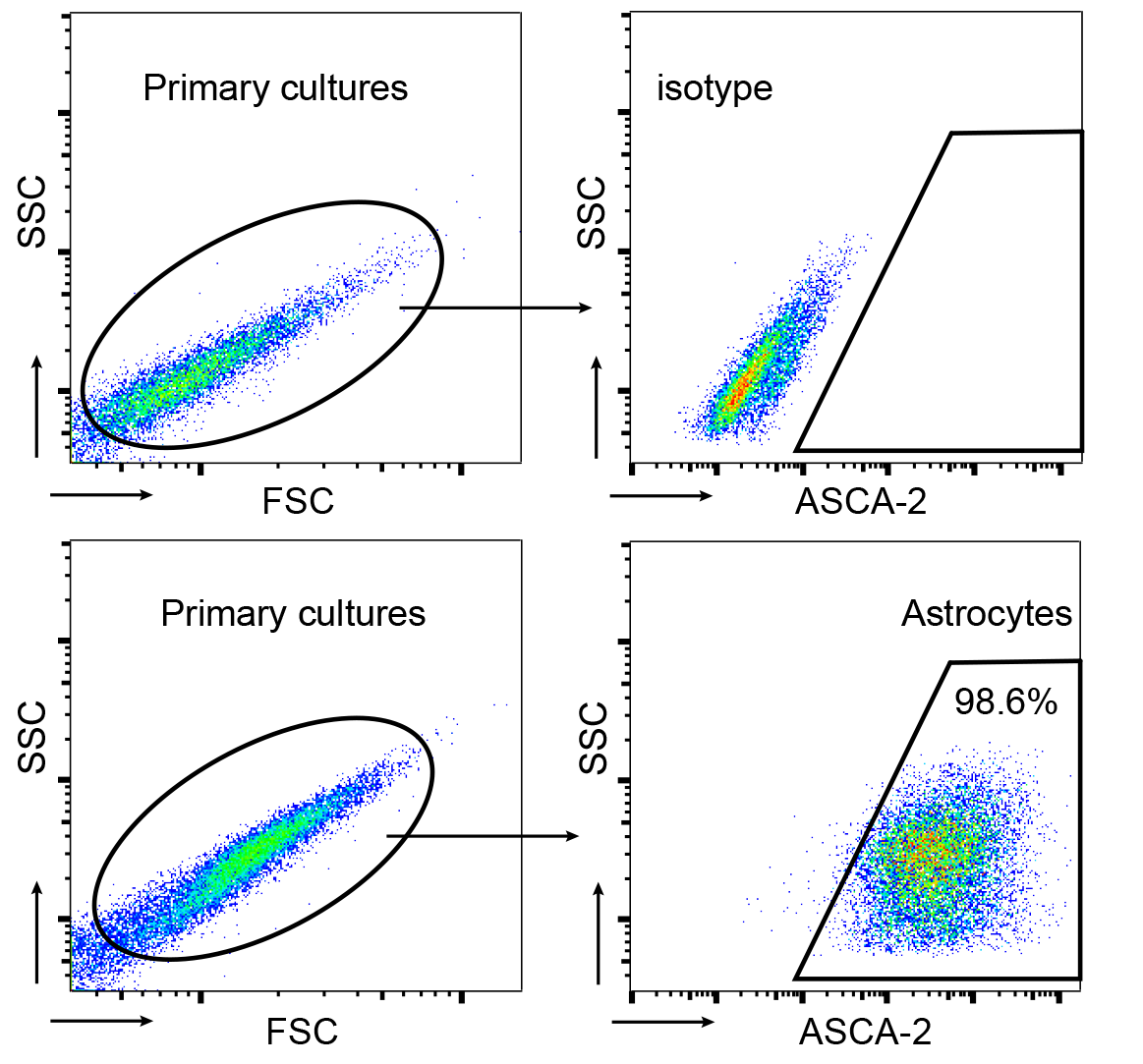

Supplement: Supplementary file 2 — Additional file 2:. Supplementary Figure 2. The purity of primary cultured astrocytes. APC-ACSA-2 was used for detecting the percentage of astrocytes in the cultured cells by flow cytometry [file 12974_2021_2201_MOESM2_ESM.tif]

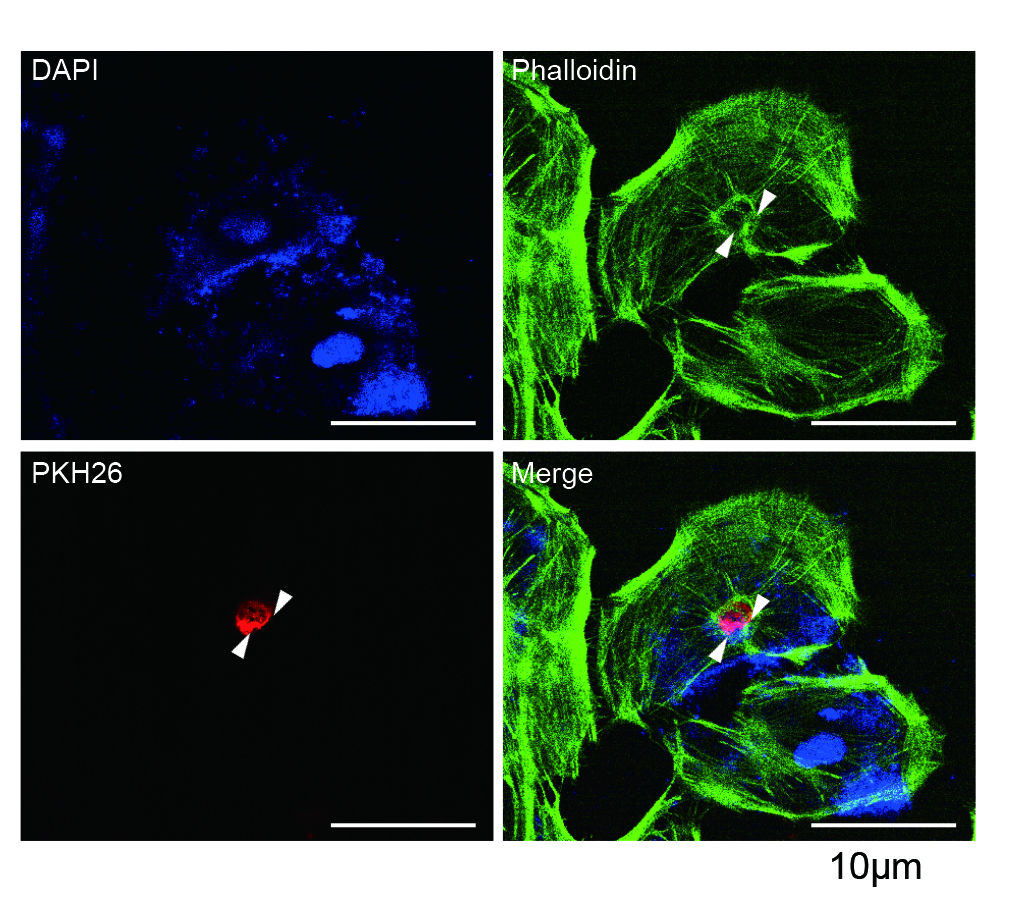

Supplement: Supplementary file 3 — Additional file 3:. Supplementary Figure 3. Representative images of phagocytic cup during astrocytic phagocytosis. The engulfed PKH26 labeled neuron (red) was surrounding by abundant F-actin (arrowhead) which is visualized by Phalloidin (green). Scale bar = 10μm [file 12974_2021_2201_MOESM3_ESM.tif]

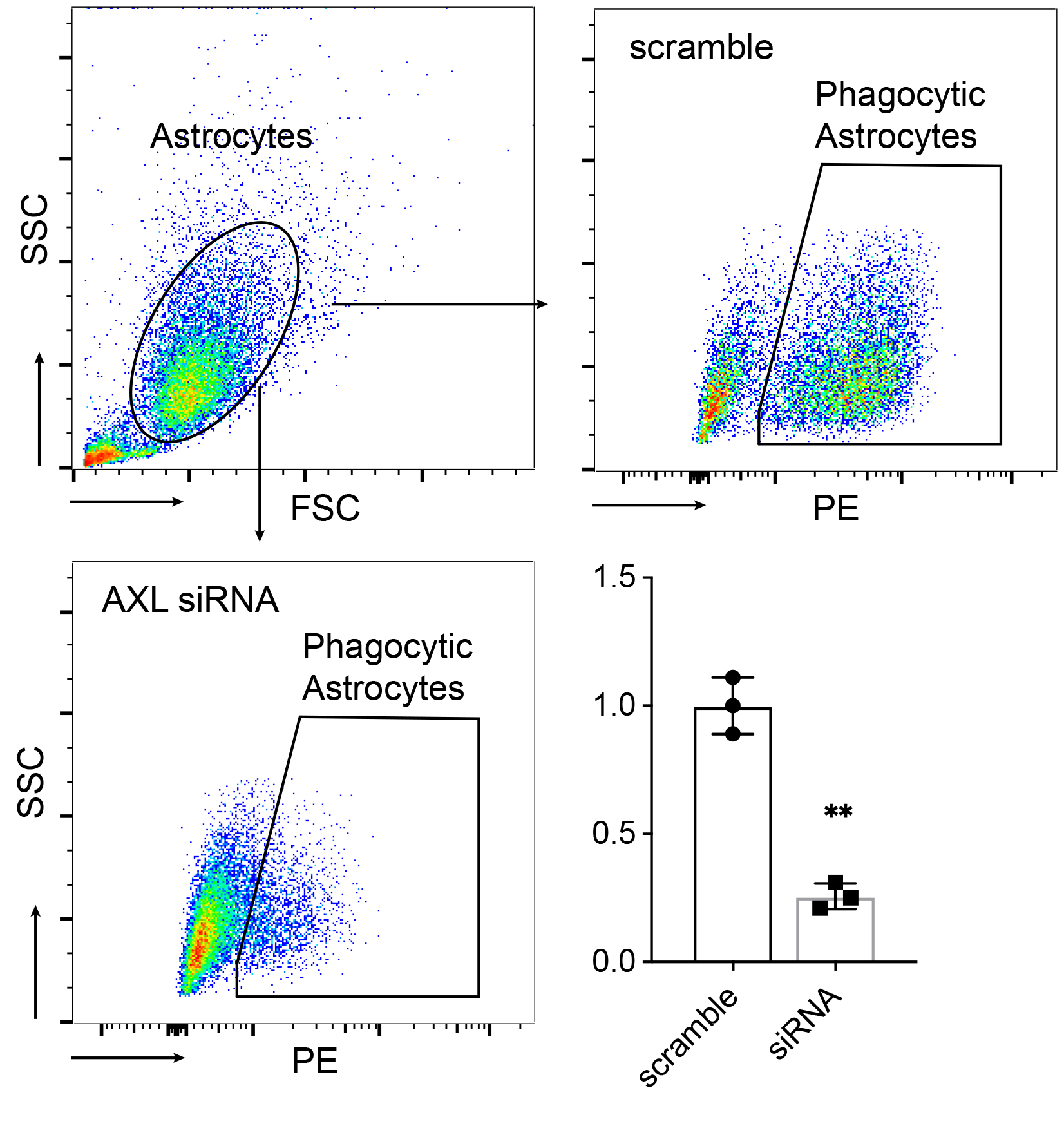

Supplement: Supplementary file 4 — Additional file 4:. Supplementary Figure 4. The astrocytic phagocytosis was assessed by flow cytometry after 24 h co-culture. Astrocytes were pretreated with AXL-siRNA or scramble siRNA for 48h prior to the addition of PKH26 labeled neurons. n=3 per group. **p<0.01 by two-tail Student’s t-test [file 12974_2021_2201_MOESM4_ESM.tif]
