# Supplementary material for: AXL kinase-mediated astrocytic phagocytosis modulates outcomes of traumatic brain injury
Source: J Neuroinflammation. 2021 Jul 7;18:154. doi: 10.1186/s12974-021-02201-3 (PMC8264993; doi:10.1186/s12974-021-02201-3)
Supplement: Supplementary file 5 — Additional file 5:. Supplementary Information: text summary [file 12974_2021_2201_MOESM5_ESM.docx]

**Supplementary Information: text summary**

Supplementary Information 1: Supplementary Text S1. mNSS protocols

Supplementary Information 2: Supplementary Figure 1. Immunofluorescence staining of GFAP in whole brain section at 3 days post TBI. The reactive astrocytes were detected in the ipsilateral section instead of the contralateral area. Scale bar = 10μm.

Supplementary Information 3: Supplementary Figure 2. The purity of primary cultured astrocytes. APC-ACSA-2 was used for detecting the percentage of astrocytes in the cultured cells by flow cytometry.

Supplementary Information 4: Supplementary figure 3. Representative images of phagocytic cup during astrocytic phagocytosis. The engulfed PKH26 labeled neuron (red) was surrounding by abundant F-actin (arrowhead) which is visualized by Phalloidin (green). Scale bar = 10μm.

Supplementary Information 5: Supplementary figure 4. The astrocytic phagocytosis was assessed by flow cytometry after 24 h co-culture. Astrocytes were pretreated with AXL-siRNA or scramble siRNA for 48h prior to the addition of PKH26 labeled neurons. n=3 per group. **p<0.01 by two-tail Student’s t-test.
