# Supplementary material for: AXL kinase-mediated astrocytic phagocytosis modulates outcomes of traumatic brain injury
Source: J Neuroinflammation. 2021 Jul 7;18:154. doi: 10.1186/s12974-021-02201-3 (PMC8264993; doi:10.1186/s12974-021-02201-3)
Supplement: Supplementary file 6 — Additional file 6:. Supplementary table 1. mNSS protocols [file 12974_2021_2201_MOESM6_ESM.docx]

Supplementary Table S1 mNSS Score.

| mNSS experimental paremeters |  | Score |
| --- | --- | --- |
| Motor tests |  |  |
| Raising mice by the tail |  |  |
| Flexion of forelimb |  | 1 |
| Flexion of hindlimb |  | 1 |
| Head deviating from the vertical axis by > 10° within 30s |  | 1 |
| Placing mice on the floor |  |  |
| Normal walking |  | 0 |
| Incapability to walk straight |  | 1 |
| Turning to the paralyzed side |  | 2 |
| Falling down to the paralyzed side |  | 3 |
| Sensory tests |  |  |
| Placement test (visual and tactile test) |  | 1 |
| Proprioception test (squeezing the claws to the table  edge to stimulate the limb muscles) |  | 2 |
| Balance beam tests |  |  |
| Stable balance posture |  | 0 |
| Grasping edge of the beam |  | 1 |
| Holding the beam, one limb falling from the beam |  | 2 |
| Holding the beam and two limbs falling from the beam   or rotating on the beam (> 60 s) |  | 3 |
| Trying to balance on the beam but falling (> 40 s) |  | 4 |
| Trying to balance on the beam but falling (> 20 s) |  | 5 |
| Trying to balance on the beam but falling (< 20 s) |  | 6 |
| Reflexes absent and abnormal movements |  |  |
| Auricle refelx (shaking head when touching the external   auditory canal) |  | 1 |
| Corneal reflex (blinking when tapping the cornea with   cotton) |  | 1 |
| Panic reflex (motor response to the noise from snapping   a cardborad) |  | 1 |
| Epilepsy, myoclonus, dystonia |  | 1 |
| Total scores |  | 18 |
| mNSS: Modified Neurological Severity Score; s: second |  |  |
